# WILDkCAT: extract, retrieve, and predict enzyme turnover numbers of constraint-based metabolic models

**DOI:** 10.1093/bioinformatics/btag510

**Published:** 2026-07-11

**Authors:** Hugues Escoffier, Carole L Linster, Thomas Sauter

**Affiliations:** Department of Health, Medicine and Life Sciences (DHML), University of Luxembourg, Esch-sur-Alzette L-4362, Luxembourg; Luxembourg Centre for Systems Biomedicine (LCSB), University of Luxembourg, Esch-sur-Alzette L-4362, Luxembourg; Department of Health, Medicine and Life Sciences (DHML), University of Luxembourg, Esch-sur-Alzette L-4362, Luxembourg

## Abstract

**Summary:**

Accurate enzyme turnover numbers are essential for building enzyme-constrained genome-scale metabolic models. However, collecting and curating these parameters remains a major bottleneck. Indeed, $k_{\mathrm{cat}}$ values are scattered across multiple databases, reported under varying experimental conditions, and often missing for many enzymes. To address this challenge, we present WILDkCAT, a Python-based pipeline that enables the retrieval of $k_{\mathrm{cat}}$ values from wild-type enzyme measured under user-specified pH and temperature ranges for a given metabolic model. The application to *Escherichia coli* (iML1515) and *Homo sapiens* (Human-GEM) models demonstrated the ability of WILDkCAT to retrieve substantial $k_{\mathrm{cat}}$ coverage and its applicability across diverse genome-scale models.

**Availability and implementation:**

WILDkCAT is available at https://github.com/sysbiolux/WILDkCAT and from PyPI. WILDkCAT works on all major operating systems and computer architectures. The documentation is available at https://sysbiolux.github.io/WILDkCAT.

## 1 Background

Genome-scale metabolic models (GEMs) are particularly powerful tools for analysing and understanding the cellular metabolism of organisms (Chen *et al*. 2019, Fang *et al*. 2020). However, standard flux balance analysis (FBA) may face optimization issues due to the lack of sufficient biological constraints (Machado and Herrgård 2014). To address this limitation, quantitative flux prediction has recently become an area of interest, resulting in significant improvements in phenotype prediction through the integration of kinetic parameters into the model (Li *et al*. 2022, Kratochvíl *et al*. 2025). Several methods (Bekiaris and Klamt 2020, Chen *et al*. 2024, Kratochvíl *et al*. 2025) have been developed over time to enable the reconstruction of more realistic models by incorporating kinetics as an additional constraint.

Experimentally measured turnover numbers ($k_{\mathrm{cat}}$) are sparse and variable. They are typically obtained from *in vitro* enzyme assays, which are processes that are dependent on numerous parameters, including pH, temperature, and substrate (Davidi *et al*. 2016). These parameters can substantially influence catalytic rates, thereby limiting the availability of $k_{\mathrm{cat}}$ measurements tailored to conditions or organisms. Thus, genome-wide modelling approaches typically adopt ‘fuzzy matching’ strategies to recover $k_{\mathrm{cat}}$ values from homologous enzymes or related organisms (Bekiaris and Klamt 2020, Lewis *et al*. 2021, Chen *et al*. 2024).

Existing tools automate the retrieval of enzymatic parameters. For example, the GECKO method (Chen *et al*. 2024) includes a hierarchical, automated query to the experimental database BRENDA (Chang *et al*. 2021) yielding broad coverage for yeast. The GECKO 3.0 method (Chen *et al*. 2024) refines this with a phylogenetic matching step: if no exact organism match, the method prioritises the $k_{\mathrm{cat}}$ measurements from the phylogenetically closest organism. The COBRA-k pipeline (Bekiaris and Klamt 2026) queries the two most comprehensive enzyme kinetics databases BRENDA (Chang *et al*. 2021) and SABIO-RK (Wittig *et al*. 2018) and prefers substrate-specific values from the same organism (or nearest species) and otherwise uses averages. These approaches increase coverage but can assign imprecise values when exact matches are lacking. Moreover, recent deep learning methods also address missing $k_{\mathrm{cat}}$ values. DLKcat (Li *et al*. 2022) and CataPro (Wang *et al*. 2025) use deep learning on enzyme sequences and substrates to predict $k_{\mathrm{cat}}$. These tools offer fallback values when experimental data are unavailable.

In this work, we present the WILDkCAT pipeline that facilitates the retrieval of $k_{\mathrm{cat}}$ values for a given metabolic model. The present approach uses a penalty-based system to rank the closest $k_{\mathrm{cat}}$ values based on reaction, organism, and experimental conditions specified by the user (pH and temperature). Moreover, WILDkCAT also incorporates the Arrhenius equation as a temperature correction tool. All information is stored in a TSV file containing all the selected $k_{\mathrm{cat}}$ metadata, allowing for the user a complete data traceability. In addition, an HTML report containing the results and quality control for each step of the pipeline is generated.

In this publication, WILDkCAT was used to retrieve $k_{\mathrm{cat}}$ values under physiological temperature and pH conditions for both *Escherichia coli* (iML1515 model (Monk *et al*. 2017)) and *Homo sapiens* (Human-GEM (Luo *et al*. 2026)), demonstrating its applicability across different organisms and genome-scale metabolic models.

## 2 Implementation

**Figure 1:**
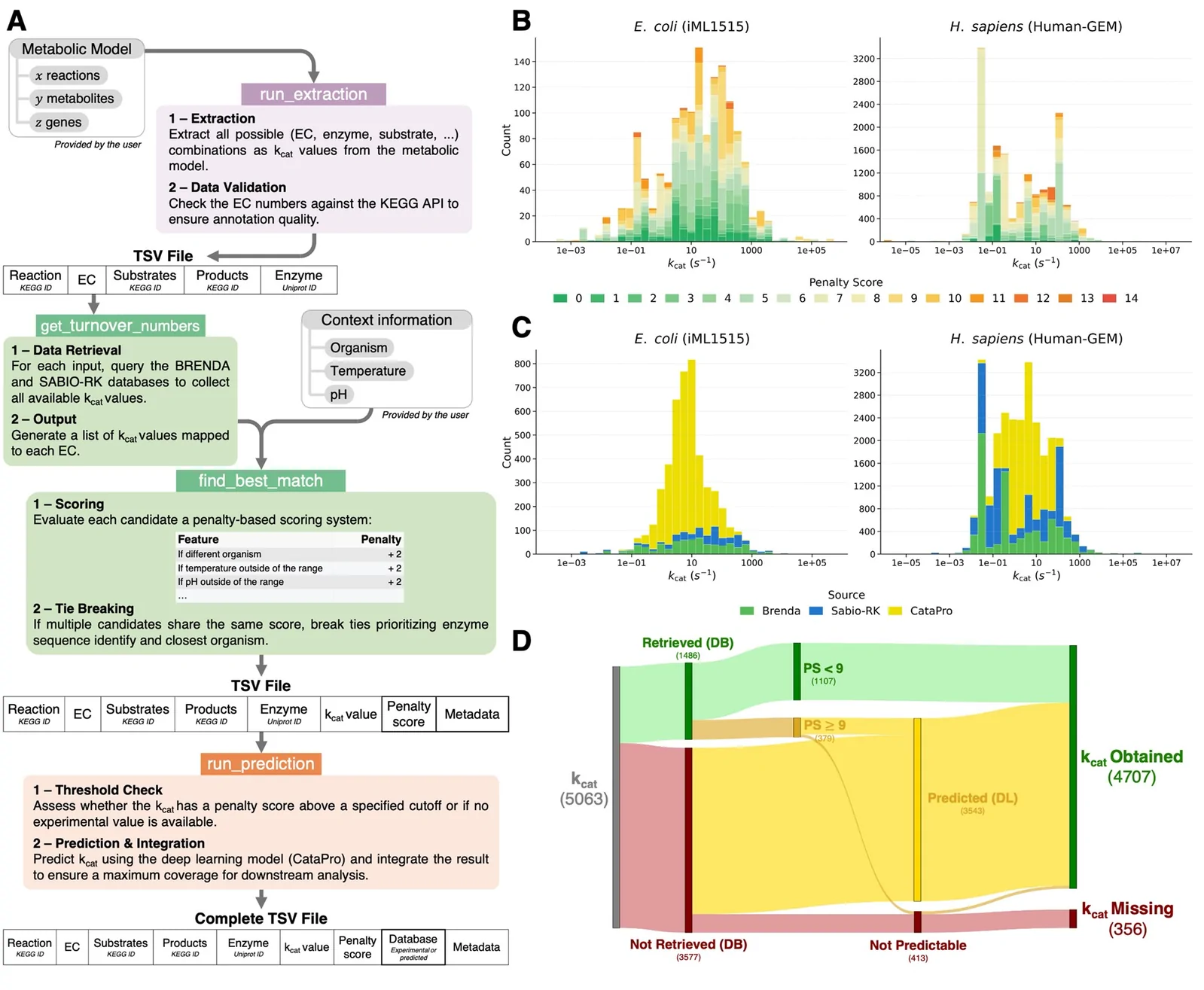
(A) Simplified workflow of WILDkCAT. The workflow consists of three main steps: extraction (purple), retrieval (green), and prediction (orange). The key functions associated with each step are shown in coloured boxes; grey boxes indicate the user-provided inputs. (B) Distribution of experimentally retrieved $k_{\mathrm{cat}}$ values for the iML1515 and Human-GEM models, with stacked bars indicate penalty scores (0–14) quantifying the mismatch between the experimental entry and the target enzyme-reaction pair, with 0 reflecting an exact match and higher values representing increasing deviations in substrate specificity, enzyme isoform, organism, temperature, and pH. (C) Distribution of $k_{\mathrm{cat}}$ values for iML1515 and Human-GEM stratified by data source (BRENDA, SABIO-RK or CataPro predictions) showing the contributions of experimental and predicted sources. (D) Sankey plot summarizing the proportion of enzyme-reaction combinations for which $k_{\mathrm{cat}}$ values were obtained through the WILDkCAT pipeline. The width of each flow is proportional to the number of combinations in each category. DB, experimental database; PS, penalty score; DL, Deep learning prediction.

WILDkCAT is a command-line-interface (CLI) tool designed to retrieve the $k_{\mathrm{cat}}$ values for a given metabolic model. In addition to the CLI, a programmatic access is also available and documented. The pipeline accepts as input a given metabolic model in the following formats: object-oriented JSON, SBML, and COBRA models saved in a MAT file. SBML is the recommended format for interoperability and reproducibility, as it is the primary community standard for systems biology model exchange. A schematic representation of the pipeline is given in Fig. 1A.

First, the pipeline extracts information for all the enzyme-reaction combinations present in the model. These combinations are stored in a TSV file, with each $k_{\mathrm{cat}}\;$value represented on a separate line. When available, WILDkCAT also retrieves the Enzyme Commission number (EC number) associated with the reaction. EC numbers correspond to an identifiers that classify enzymes based on the chemical reactions they catalyse (McDonald and Tipton 2023). By incorporating this additional information, the search can be expanded to include homologous enzymes with the same EC number, thereby providing a fallback to maximise coverage.

Second, WILDkCAT systematically queries the two most comprehensive enzyme kinetics databases, BRENDA (Chang *et al*. 2021) and SABIO-RK (Wittig *et al*. 2018), to retrieve experimentally determined $k_{\mathrm{cat}}$ values from wild-type enzymes. The retrieved entries are then subjected to a scoring system that penalises mismatches in substrate, enzyme isoform, organism, temperature, and pH, thus ensuring that conditions that more closely resemble those of the target enzyme are given priority. This penalty system allows to give a higher priority to values measured within user-specified pH and temperature ranges (Fig. S4, available as supplementary data at *Bioinformatics* online), enabling context-relevant $k_{\mathrm{cat}}$ selection when multiple entries are available.

When experimental measurements are available at multiple temperatures and thus allow estimation of the activation energy (*E_a_*), $k_{\mathrm{cat}}$ values are temperature-corrected using the Arrhenius equation (Jensen 1985). This correction is applied if at least two temperature measurements exist to fit the Arrhenius relationship (Supplementary System 2, available as supplementary data at *Bioinformatics* online). When penalty scores are tied, values are ranked by sequence identity and phylogenetic proximity, remaining ties are broken by selecting the highest $k_{\mathrm{cat}}$ value. Through this combined strategy of penalization, ranking, and correction, the pipeline ensures that the selected $k_{\mathrm{cat}}$ values are as accurate and comparable as possible for integration into the model. Details are provided in Supplementary Section S1, available as supplementary data at *Bioinformatics* online. Subsequently, different EC numbers associated with the same combination of enzyme and reaction are merged to avoid redundant enzyme-reaction mappings, thereby reducing the number of combinations to a nonredundant set.

Third, WILDkCAT optionally enables the use of CataPro (Wang *et al*. 2025), the current state-of-the-art predictive model for $k_{\mathrm{cat}}$ estimation, to fill gaps where no suitable experimental values are found. Predictions are only used if the penalty score of the retrieve value is higher a user-specified threshold, thus ensuring that experimental data are always prioritised. This hybrid strategy provides a comprehensive and reliable $k_{\mathrm{cat}}$ dataset tailored to the model of interest.

WILDkCAT provides a TSV file containing all $k_{\mathrm{cat}}$ values with their source (experimental or predicted), their penalty score and all the corresponding metadata as final output. This allows the user to determine the origin and measurement conditions of each value assigned by the pipeline.

At the end of each stage of the process (extraction, retrieval, and prediction), an HTML report is generated. The purpose of these documents is to summarise the obtained results through figures and tables, to assess the quality of the corresponding step.

## 3 Selected use-cases

### 3.1 Retrieval of $k_{\mathrm{cat}}\;$values for the iML1515 model of *Escherichia coli*

To demonstrate the applicability of the WILDkCAT pipeline, it was used to retrieve $k_{\mathrm{cat}}$ values for the *Escherichia coli* GEM model iML1515 (2712 reactions, 1877 metabolites and 1516 genes) (Monk *et al*. 2017). In the first stage, the pipeline extracted all possible enzyme-reaction combinations from the model, resulting in 5063 unique pairs covering 2023 reactions out of the 2712 reactions included in iML1515.

In the second stage, using the BRENDA (Chang *et al*. 2021) and SABIO-RK (Wittig *et al*. 2018) APIs, $k_{\mathrm{cat}}$ values were successfully retrieved for 29.4% of the enzyme-reaction combinations. The desired temperature and pH ranges were chosen to prioritize physiologically relevant conditions, temperatures between 20–45°C and pH values between 4–8. Each retrieved value was associated with a penalty score ranging from 0 to 13 (Supplementary System 1, available as supplementary data at *Bioinformatics* online), reflecting the degree of confidence in the experimental match (Fig. 1B).

In the final stage, combinations lacking experimental $k_{\mathrm{cat}}\;$values or having penalty scores above a threshold of nine (corresponding to 78.1% of the dataset) were predicted via the CataPro (Wang *et al*. 2025) deep learning model. A threshold of nine was chosen as it marks the boundary above which retrieved values deviate from both the target organism and substrate, making deep learning predictions preferable (Supplementary Section S1, available as supplementary data at *Bioinformatics* online).

Among these, 80.1% could be successfully predicted. At the end of the pipeline across the 5063 unique pairs covering 2023 reactions of the model, 4707 have an assigned $k_{\mathrm{cat}}$ value (retrieved from experimental database or predicted) (Fig. 1C and D) covering 1907 reactions. The proportion of reactions with experimentally determined $k_{\mathrm{cat}}$ values relative to the predicted reactions varies across pathways. Notably, reactions implicated in the amino acid metabolism are more frequently retrieved from experimental databases, whereas transport reactions are predominantly predicted (Fig. S3, available as supplementary data at *Bioinformatics* online).

### 3.2 Retrieval of $k_{\mathrm{cat}}\;$values for the human-GEM model of *Homo-sapiens*

To further evaluate the scalability and versatility of the pipeline, WILDkCAT was also applied to Human-GEM (v.2.0.0) (Luo *et al*. 2026), corresponding to a more complex organism model (12,931 reactions, 8461 metabolites and 2848 genes) (Robinson *et al*. 2020). Using the same procedure described for *E. coli*, 28,461 enzyme-reaction combinations were identified, covering 7804 reactions. Experimental $k_{\mathrm{cat}}$ values were retrieved for 59.9% of these pairs with penalty scores ranging from 0 to 14, for temperature preferences between 36–38°C and pH preferences between 7–8, which is consistent with physiological ranges in humans (Fig. 1B). For the remaining combinations, CataPro (Wang *et al*. 2025) predicted $k_{\mathrm{cat}}$ values lacking of experimental values or having a penalty score above nine, resulting in a retrieval 27 552 $k_{\mathrm{cat}}$ values covering 7533 reactions.

Human-GEM achieved higher experimental coverage than iML1515 (59.9% vs. 29.4% and 53.6% vs. 23% for high-confidence assignments with penalty score < 9). Nevertheless, by combining experimental retrieval with deep learning predictions, WILDkCAT was able to assign $k_{\mathrm{cat}}$ values to a substantial fraction of reactions (above 90% in both models), demonstrating its applicability to large and complex metabolic networks.

## 4 Discussion

WILDkCAT provides reproducible $k_{\mathrm{cat}}$ retrieval and prediction under user-specified conditions, with full data traceability and a penalty score serving as a confidence metric. Moreover, as shown previously, the WILDkCAT pipeline is applicable to different models representing diverse organisms and developed by various laboratories, thereby illustrating the versatility of the approach.

Thus, WILDkCAT represents a pivotal element in the development of enzyme-constrained models, thereby eliminating a significant impediment to their large-scale development and application.

In the future, further improvements in $k_{\mathrm{cat}}$ retrieval could be achieved through the adoption of more advanced API systems by major biochemical databases BRENDA (Chang *et al*. 2021) and SABIO-RK (Wittig *et al*. 2018), facilitating more efficient and standardized data access. Furthermore, the consistent utilisation of unambiguous chemical identifiers, such as KEGG identifiers (Kanehisa and Goto 2000) or simplified molecular input line entry system (SMILES), would help to avoid issues related to metabolite name matching. Finally, WILDkCAT could be extended to retrieve additional kinetic parameters, such as Michaelis-Menten constants (*K_m_*) and inhibition constant (*K_i_*), which are valuable for the construction of ordinary differential equation-based and other kinetic models (Link *et al*. 2014).

## Supplementary Material

btag510_Supplementary_Data

## Data Availability

Data and scripts that replicate the results are available from https://doi.org/10.5281/zenodo.17856671.

## References

[btag510-B1] Bekiaris PS , Klamt S. Automatic construction of metabolic models with enzyme constraints. BMC Bioinform 2020;21:19. 10.1186/s12859-019-3329-9. PMC6961255 31937255

[btag510-B2] Bekiaris PS , Klamt S. COBRA-k: a powerful framework bridging constraint-based and kinetic metabolic modeling. Sci Adv 2026;12:eaeb3022. 10.1126/sciadv.aeb3022. 41564185 PMC12822651

[btag510-B3] Chang A , Jeske L, Ulbrich S et al BRENDA, the ELIXIR core data resource in 2021: new developments and updates. Nucleic Acids Res 2021;49:D498–508. 10.1093/nar/gkaa1025. 33211880 PMC7779020

[btag510-B4] Chen Y , Gustafsson J, Tafur Rangel A et al Reconstruction, simulation and analysis of enzyme-constrained metabolic models using GECKO toolbox 3.0. Nat Protoc 2024;19:629–67. 10.1038/s41596-023-00931-7. 38238583

[btag510-B5] Chen Y , Li G, Nielsen J. Genome-scale metabolic modeling from yeast to human cell models of complex diseases: latest advances and challenges. Methods Mol Biol 2019;2049:329–45. 10.1007/978-1-4939-9736-7_19. 31602620

[btag510-B6] Davidi D , Noor E, Liebermeister W et al Global characterization of in vivo enzyme catalytic rates and their correspondence to in vitro kcat measurements. Proceedings of the National Academy of Sciences of the United States of America 2016;113:3401–6. 10.1073/pnas.1514240113. 26951675 PMC4812741

[btag510-B7] Fang X , Lloyd CJ, Palsson BO. Reconstructing organisms in silico: genome-scale models and their emerging applications. Nat Rev Microbiol 2020;18:731–43. 10.1038/s41579-020-00440-4. 32958892 PMC7981288

[btag510-B8] Jensen F. Activation energies and the Arrhenius equation. Qual Reliabil Eng 1985;1:13–7. 10.1002/qre.4680010104.

[btag510-B9] Kanehisa M , Goto S. KEGG: Kyoto encyclopedia of genes and genomes. Nucleic Acids Res 2000;28:27–30. 10.1093/nar/28.1.27. 10592173 PMC102409

[btag510-B10] Kratochvíl M , Wilken SE, Ebenhöh O et al COBREXA 2: tidy and scalable construction of complex metabolic models. Bioinformatics 2025;41:btaf056. 10.1093/bioinformatics/btaf056. 39921902 PMC11842047

[btag510-B11] Lewis JE , Forshaw TE, Boothman DA et al Personalized genome-scale metabolic models identify targets of redox metabolism in radiation-resistant tumors. Cell Syst 2021;12:68–81.e11. 10.1016/j.cels.2020.12.001. 33476554 PMC7905848

[btag510-B12] Li F , Yuan L, Lu H et al Deep learning-based kcat prediction enables improved enzyme-constrained model reconstruction. Nat Catal 2022;5:662–72. 10.1038/s41929-022-00798-z.

[btag510-B13] Li G-H , Han F, Xiao F-H et al System‐level metabolic modeling facilitates unveiling metabolic signature in exceptional longevity. Aging Cell 2022;21:e13595. 10.1111/acel.13595. 35343058 PMC9009231

[btag510-B14] Link H , Christodoulou D, Sauer U. Advancing metabolic models with kinetic information. Curr Opin Biotechnol 2014;29:8–14. 10.1016/j.copbio.2014.01.015. 24534671

[btag510-B15] Luo J , Wang H, Moyer D et al Reconstruction of human metabolic models with large language models. Proc Natl Acad Sci U S A 2026;123:e2516511123. 10.1073/pnas.2516511123. 41950094 PMC13079975

[btag510-B16] Machado D , Herrgård M. Systematic evaluation of methods for integration of transcriptomic data into constraint-based models of metabolism. PLoS Comput Biol 2014;10:e1003580. 10.1371/journal.pcbi.1003580. 24762745 PMC3998872

[btag510-B17] McDonald AG , Tipton KF. Enzyme nomenclature and classification: the state of the art. Febs J 2023;290:2214–31. 10.1111/febs.16274. 34773359

[btag510-B18] Monk JM , Lloyd CJ, Brunk E et al iML1515, a knowledgebase that computes *Escherichia coli* traits. Nat Biotechnol 2017;35:904–8. 10.1038/nbt.3956. 29020004 PMC6521705

[btag510-B19] Robinson JL , Kocabaş P, Wang H et al An atlas of human metabolism. Sci Signal 2020;13:eaaz1482. 10.1126/scisignal.aaz1482. 32209698 PMC7331181

[btag510-B20] Wang Z , Xie D, Wu D et al Robust enzyme discovery and engineering with deep learning using CataPro. Nat Commun 2025;16:2736. 10.1038/s41467-025-58038-4. 40108140 PMC11923063

[btag510-B21] Wittig U , Rey M, Weidemann A et al SABIO-RK: an updated resource for manually curated biochemical reaction kinetics. Nucleic Acids Res 2018;46:D656–60. 10.1093/nar/gkx1065. 29092055 PMC5753344

