# Supplementary material for: WILDkCAT: extract, retrieve, and predict enzyme turnover numbers of constraint-based metabolic models
Source: Bioinformatics. 2026 Jul 11;42(8):btag510. doi: 10.1093/bioinformatics/btag510 (PMC13450424; doi:10.1093/bioinformatics/btag510)
Supplement: Supplementary file 1 [file btag510_supplementary_data.docx]

**Supplementary Materials**

**S1. WILDkCAT Pipeline**

**S1.1. Extraction based on the annotations available in the model**

The pipeline accepts as input a given metabolic model in the multiple formats: object-oriented JSON, SBML, and COBRA models saved in a MAT file. All formats are treated equivalently once loaded into a COBRApy `cobra.Model` object. MAT file support is provided via COBRApy's `load_matlab_model()` function.

During the first step, $k_{cat}$-related information is extracted from the annotations available in the model. In addition to the reaction, metabolite, and gene information, the fields checked during the extraction included ‘ec-code’ and ‘kegg.reaction’ for the reactions, ‘kegg.compound’ for the metabolites and ‘uniprot’ for the genes [1,2]. An example of this extraction process is shown in Supplementary Figure 1.

During this step, each EC number is validated against the KEGG database [1]. The transferred EC number are reported in a log file, indicating, if they exist, the EC number(s) to which it has been transferred.

The EC numbers that are incomplete (*e.g.* 1.1.-.-) or in an incorrect format (*e.g.* 11125) are reported in a log file. These cases are also flagged in the ‘warning_ec’ column as follows:

- ‘transferred’: the EC number provided have been transferred
- ‘incomplete’: the EC number provided is written in an incorrect format
- ‘none’: no EC number is provided for this combination

For the reactions catalysed by multiple enzymes, corresponding to an AND in the Gene-Protein-Reaction (GPR) rules, the multiple enzymes are treated as an enzymatic complex and the catalytic enzyme of this complex is identified using the UniProt database [2]. When a single enzyme is associated with a reaction, it assumed to be the catalytic enzyme. These cases are flagged in the ‘warning_enz’ column as follows:

- empty: a single catalytic enzyme is identified
- ‘none’: no catalytic enzyme could be identified
- ‘multiple’: multiple enzymes were identified
- ‘no_gpr’: the corresponding reaction has no GPR rules in the model.

Finally, all the extracted data are stored in a TSV file. Rows without a valid EC number (*i.e.* missing, incorrect or transferred) and without an identifiable catalytic enzyme (*i.e.* none, multiple or no_gpr are removed, as they contain insufficient information for subsequent retrieval.

For $k_{cat}$ retrieval to proceed, at least one of the following must be present: a valid EC number (ec-code) associated with the reaction, or a GPR rule. For $k_{cat}$ prediction, both a GPR rule and a metabolite with a KEGG identifier are required. The recommended and mandatory information are recapitulated in Supplementary Table 1. The impact of missing annotations is reported in the report ‘extraction_report.html’.

WILDkCAT does not define a minimum annotation coverage threshold, as the appropriate level depends on the specific application and model. When annotation coverage is limited, the number of reactions for which $k_{cat}$ values can be assigned decreases proportionally. Users working with poorly annotated models are therefore encouraged to use dedicated annotation tools such as CobraMod [3] prior to running WILDkCAT. Beyond overall coverage, pathway-level completeness should also be considered, as missing $k_{cat}$ values in specific pathways may impair downstream analyses of those pathways even when global coverage appears sufficient.

**S1.2. Retrieval of the corresponding** $\boldsymbol{k}_{\boldsymbol{cat}}$ **values in experimental databases**

Depending on the data available, WILDkCAT retrieves the ‘closest possible match’ for each $k_{cat}$ value, corresponding to the experimental measurements obtained under the most similar conditions to those specified by the user.

To do this, a penalty-based system is used, which is mathematically defined in Supplementary System 1.

The penalty score quantifies how closely a candidate $k_{cat}$ entry matches the query enzyme and experimental conditions requested by the user. A score of zero indicates an exact match whereas a score of 14 indicates that the selected $k_{cat}$ value is exclusively sharing the EC number with the query enzyme. A score of 15 corresponds to cases where only mutant enzyme values are available and a score of 16 indicates that no information was found in the database.

Penalty weights are assigned according to the expected impact of each parameter on $k_{cat}$ deviation, quantified by a sensitivity analysis in which $k_{cat}$ values retrieved under exact user-defined conditions (penalty score = 0) for the iML1515 model were compared to database entries differing by exactly one parameter at a time (organism, substrate, enzyme isoform within the same organism, temperature, and pH). The resulting distributions (Supplementary Fig. 2) reveal that organism mismatch introduces the largest systematic deviation, followed by substrate and enzyme isoform, thereby supporting the assignment of higher penalty weights to these parameters. Variation in temperature and pH, although still influential, introduces comparatively smaller deviations and is therefore penalised more lightly. The penalty weights are motivated by these observed fold-change distributions. However, the precise discrete values assigned to each criterion are to some extent arbitrary. Nevertheless, all weights remain user-adjustable in the matching.py file, allowing the scoring scheme to be adapted to specific organisms, experimental contexts, or prioritisation strategies.

To further validate the penalty score as a confidence metric, the Median Absolute Error (MedAE) was computed between retrieved values and exact-match entries (penalty score = 0) across all penalty levels. As shown in Supplementary Fig. 3, MedAE exhibits a generally increasing trend with penalty score. Although the relationship is not strictly monotonic, the overall trend supports the interpretation of the penalty score as a meaningful and interpretable confidence metric.

This weighting ensures that the penalty scoring system prioritizes mechanistically meaningful differences when selecting the most appropriate $k_{cat}$ value. Finally, the penalty scores can be adjusted by the user by modifying their values in the matching.py file. This allows customization according to specific research priorities and preferred criteria for ranking candidate $k_{cat}$ values.

When a valid EC number is provided, the retrieval process allows the identification of other enzymes sharing the same EC number. In the case of multiple candidates sharing identical penalty scores, the closest enzyme is selected based on sequence similarity using BioPython’s Align.PairwiseAligner() function [4]. If the enzyme sequence is not available, the organism with the most similar taxonomy is identified using Entrez.efetch() [4]. When ties persist, the entry with the highest $k_{cat}$ value is selected to avoid overly restrictive assumptions during model construction, as higher $k_{cat}$ values impose less stringent constraints on the resulting model.

When no valid EC is available, the retrieval is performed based solely on the enzyme name. In this case, only alternate substrates, temperature ranges, and pH ranges are used for the filtering.

Finally, if a $k_{cat}$ value is not available in the target temperature range but at least two other measurements exist at different temperatures, the pipeline estimates the $k_{cat}$ value at the center of the temperature range by applying the Arrhenius equation (Supplementary System 2). The available data are used to approximate the activation energy (*E_a_*) [5]. Although it is possible to estimate the activation energy using only two values, it should be noted that this may not be sufficient to obtain an accurate estimate. To provide transparency regarding how these values are estimated, the ‘warnig_arr’ column is left blank if no correction is applied or contains an integer corresponding to the number of values used to estimate the activation energy when the $k_{cat}$ have been corrected.

**S1.3. Prediction of missing** $\boldsymbol{k}_{\boldsymbol{cat}}$ **values using CataPro deep learning model**

Missing $k_{cat}$ values and those associated with high penalty scores are predicted via the CataPro deep learning model [6]. CataPro takes as input the amino acid sequence of the catalytic enzyme, which is retrieved using UniProt, and the SMILES of the substrate, which is retrieved using PubChem [7]. If multiple substrates are involved in the reaction, BRENDA API is queried to identify cofactors associated with the reaction. If multiple candidate substrates remain after this step the $k_{cat}$ prediction is performed for all the remaining substrates involved in the reaction, then the lowest predicted $k_{cat}$ value is retained.

If either of the amino acid sequence of the enzyme or the substrate SMILES cannot be retrieved, no prediction is performed of that $k_{cat}$ value. In this case, if an experimental value has been retrieved at the previous step, it is retained even if the associated penalty score exceeds the user-defined threshold. Otherwise, the enzyme-substrate combination remains without an assigned $k_{cat}$ value.

Regarding the prediction threshold, a threshold of nine was selected. A score of nine corresponds to the boundary above which retrieved $k_{cat}$ values necessarily deviate from both the target organism (P_Organism_ = 2 & P_Enzyme_ = 3) and the target substrate (P_Substrate_ = 4) meaning that these two parameters are mismatched. Below this threshold, it remains possible to retrieve values measured in the same organism or with the same substrate, which we consider the minimum criterion for experimental relevance. The impact of varying the threshold is a shift in the ratio of experimentally retrieved to predicted values, since values excluded by a stricter threshold are replaced by CataPro predictions when available, and retained otherwise.

After the prediction, both the predicted and retrieved $k_{cat}$ values are integrated into a TSV file. The source of each $k_{cat}$ value, whether CataPro [6], BRENDA [8] or Sabio-RK [9], is indicated in the ‘db’ column. For experimentally retrieved $k_{cat}$ values, all metadata associated with the selected $k_{cat}$ value are stored in the subsequent columns.

**S2. Comparison between WILDkCAT with other existing methods (COBRA-k, GECKO 3.0)**

Pipelines for the automated extraction of $k_{cat}$ from biochemical databases have already been implemented in two constraint-based modelling frameworks: GECKO 3.0 [10] and COBRA-k [11]. GECKO 3.0 retrieves $k_{cat}$ values from BRENDA using enzyme-substrate matching and supplements missing values using DLKcat [12]. COBRA-k queries BRENDA and SABIO-RK. To assess the quantitative performance of WILDkCAT relative to these approaches, we applied all three methods to iML1515 model.

**S2.1. Retrieval of the** $\boldsymbol{k}_{\boldsymbol{cat}}$ **values from databases**

WILDkCAT achieved to retrieve 4707 $k_{cat}$ values corresponding to a coverage of 1907 of model reactions, exceeding that of GECKO 3.0 (4404 $k_{cat}$ ; 1883 reactions) and COBRA-k (174 $k_{cat}$ ; 163 reactions). In term of proportions, GECKO 3.0 retrieve more values from experimental databases, as it always prioritizes experimental over predictions, whereas for WILDkCAT, the values with a penalty score greater than nine were predicted. (Supplementary Fig. 4).

Beyond coverage, the three methods differ in how candidate $k_{cat}$ values are selected when multiple entries exist. COBRA-k and GECKO 3.0 filters entries by substrate and source organism but does not account for assay conditions, notably pH and temperature. WILDkCAT addresses this limitation by incorporating a multi-criteria scoring scheme that penalizes entries deviating from user-specified physiological conditions and prioritize by sequence identity before when enzymes are available, yielding condition-aware $k_{cat}$ assignments.

**S2.2. Prediction of the** $\boldsymbol{k}_{\boldsymbol{cat}}$ **values using deep learning.**

For reactions lacking experimental $k_{cat}$ values, or for which retrieved values carry a high penalty score in WILDkCAT, both GECKO 3.0 and WILDkCAT provide deep learning-based predictions. GECKO 3.0 employs DLKcat, while WILDkCAT integrates CataPro. According to benchmarks reported in the original CataPro publication [6], CataPro outperforms DLKcat across all standard regression metrics, achieving an RMSE of 1.329, a Pearson correlation coefficient (PCC) of 0.497, and a Spearman correlation coefficient (SCC) of 0.495, compared to 1.426, 0.368, and 0.349 for DLKcat, respectively.

**S2.3. Comparison of the overall coverage**

Taken together, WILDkCAT provides the broadest and most condition-specific $k_{cat}$ parameterization for iML1515 among the three methods evaluated. Its multi-criteria retrieval strategy yields qualitatively more relevant values by explicitly accounting for pH, temperature and enzyme similarity parameters that are ignored by both GECKO 3.0 and COBRA-k.

**S3. Application of the WILDkCAT pipeline to use-case models**

**S3.1. Discussion of the** $\boldsymbol{k}_{\boldsymbol{cat}}$ **values obtained for the iML1515 model**

Following the retrieval and prediction of the $k_{cat}$ values for the iML1515 model [13], WILDkCAT successfully identified values for 93% of the $k_{cat}$ values, covering 70.3% of the model reactions. Among the $k_{cat}$ values predicted using the CataPro model [6], a substantial fraction corresponds to reactions without an associated EC number into the reaction in the model (Supplementary Fig. 5) and 69.2% of these reactions are transport reactions. Transport reaction represents the transfer of one metabolite from one cellular compartment to another without altering their chemical structure. Transport reactions are essential for modelling the movement of metabolites between organelles and across membranes, thereby ensuring mass balance and connectivity in the network. The enzymes catalysing these transport reactions are not represented in experimental databases in the case of the iML1515 model. We argue that $k_{cat}$ predictions for such reactions tend to be less reliable due to the limited availability of experimental data, which limits the performance of deep learning models. Consequently, it is suggested that these $k_{cat}$ values be excluded when high-confidence data are required. However, the decision was made to make these data available to provide comprehensiveness. To assist users in identifying these low-confidence predictions, a dedicated 'Warning' section is provided in the HTML prediction report, displaying the number of predicted transport reactions. The corresponding reactions are additionally listed in the 'prediction_part2' log file for detailed inspection.

It is also important to note that enzyme-constrained metabolic modelling approaches do not require complete coverage, as only a subset of reactions limit fluxes in the model. We show that the well-documented categories such as ‘Amino Acid Metabolism’ or ‘Energy Production and Conversion’, have a good proportion of reactions with experimental $k_{cat}$ values compared to other, such as ‘Transport, Outer/Inner Membrane’ (Supplementary Fig. 6). These categories include multiple subsystems from the iML1515 model. The details of each category are detailed in the description of the Supplementary Figure 6.

The distributions of the $k_{cat}$ values retrieved by the WILDkCAT pipeline were also examined. Among the $k_{cat}$ values retrieved from databases with corresponding information, 98.2% fall within the user-specified temperature range, and 83.4% fall within the appropriate pH range, highlighting that experimental data recovery is prioritised and recovered within the ranges requested by the user. The distributions of temperature and pH for the database-extracted $k_{cat}$ values for the *Escherichia coli* model are shown in Supplementary Figure 7.

**Supplementary System 1:** Penalty score for assessing the similarity between target $k_{cat}$ values and database entries. A penalty score ranging from 0 to 14 is used to quantify the similarity between the target $k_{cat}$ value and the candidate $k_{cat}$ value retrieved from reference databases. A score of zero indicates an exact match, where the reported $k_{cat}$ value was measured under identical experimental conditions, using the same catalytic enzyme, substrate, organism, variant, temperature, and pH as required. In contrast, a score of 14 reflects the maximum divergence, incorporating differences across all these parameters (enzyme identity, substrate, organism, variant, temperature, and pH).

Penalty score = P_Catalytic_enzyme_ + P_Substrate_ + P_Organism_ + P_Variant_ + P_Temperature_ + P_pH_

where:

- P_Catalytic_enzyme_ is:
  - 0 if identical
  - 3 if different or unknown
- P_Substrate_ is:
  - 0 if identical
  - 4 if different or unknown
- P_Organism_ is:
  - 0 if identical
  - 2 if different or unknown
- P_Variant_ is:
  - 0 if ‘wild-type’
  - 1 if unknown
- P_Temperature_ is:
  - 0 if identical
  - 1 if unknown
  - 2 if outside the user-specified range
- P_pH_ is:
  - 0 if identical
  - 1 if unknown
  - 2 if outside the user-specified range

**Supplementary System 2:** The Arrhenius equation is used to adjust $k_{cat}$ values when they are measured at temperatures outside the user-defined temperature range, but measurements are available at other temperatures. The variables in the equation are the activation energy (*E_a_*), the universal gas constant (*R* = 8.314 J/(mol K)), the temperature at which $k_{cat}$ was measured (*T_db_*) and the temperature midpoint of the user-specified temperature range (*T_opt_*), both in Kelvin. The corrected $k_{cat}$ at the target temperature (*kcat_opt_*), is calculated from the reported value (*kcat_db_*) using the Arrhenius equation.

$${kcat}_{opt}={kcat}_{db}\times exp(-\frac{E_{a}}{R}(\frac{1}{T_{db}}-\frac{1}{T_{opt}}))$$

**Supplementary Figure 1:** Example of WILDkCAT extraction step. The top panel shows the model reaction entry (NDPK5), including formula (ATP + dGDP ⬄ ADP + dGTP) and the associated gene-protein-reaction (GPR) rule. The bottom panel illustrates how WILDkCAT stores the information into a structured TSV format, generating one row per enzyme-reaction combination. A colour code highlights the origin of each data field. Columns shown are not exhaustive, the full TSV also includes KEGG identifiers and warning flags used for downstream retrieval and quality control of the data.

**Supplementary Figure 2:** Distribution of log_10_ fold changes in $k_{cat}$ values associated with single-parameter mismatches relative to exact-match entries (penalty score = 0) in the iML1515 model. For each parameter : organism, substrate, temperature, pH, and enzyme isoform (within the same organism), $k_{cat}$ values retrieved under exact user-defined conditions were compared to database entries differing by exactly one parameter at a time. Violin plots show the full distribution of log₁₀($k_{cat}^{\mathrm{mismatch}}$/$k_{cat}^{\mathrm{perfect}}$) for each mismatch category, with individual data points overlaid as a jittered scatter plot. The dashed horizontal line at zero indicates no deviation from the exact-match reference.

**Supplementary Figure 3:** Median Absolute Error (MedAE) of log_10_($k_{cat}$) as a function of penalty score, computed between retrieved $k_{cat}$ values and exact-match entries (penalty score = 0) across all penalty levels (1-14), in the iML1515 model. MedAE exhibits a generally increasing trend with penalty score, ranging from 0.28 (penalty score = 2) to 1.12 (penalty score = 13), supporting the interpretation of the penalty score as a meaningful confidence metric for the quality of retrieved $k_{cat}$ values.

**Supplementary Figure 4:** Comparison of $k_{cat}$ retrieval coverage across COBRA-k, GECKO 3.0 and WILDkCAT applied to the iML1515 model. (A) Total number of $k_{cat}$ values assigned by each method, stratified by source (experimentally retrieved or predicted). COBRA-k retrieves a substantially lower number of experimental values and does not incorporate a prediction step. GECKO 3.0 and WILDkCAT achieve comparable total counts, with WILDkCAT yielding a higher number of assigned values overall. (B) Number of model reactions covered by at least one assigned $k_{cat}$ value. COBRA-k covers fewer than 250 reactions, whereas both GECKO 3.0 and WILDkCAT achieve coverage of 1883 and 1907 reactions respectively.

**Supplementary Figure 5:** Pie charts illustrating the coverage of $k_{cat}$ values in the iML1515 metabolic model. (A) Coverage across all enzyme-reaction associations in the model. (B) Coverage restricted to enzyme-reaction pairs annotated with an EC number. (C) Coverage for enzyme-reaction pairs lacking EC number annotation in the model.

**Supplementary Figure 6:** Distribution of reactions according to the source of $k_{cat}$ values across three pathway categories: Amino Acid Metabolism, Energy Production and Conversion, and Transport, Outer/Inner Membrane. The Amino Acid Metabolism category includes the following iML1515 subsystems: Alanine and Aspartate Metabolism, Arginine and Proline Metabolism, Cysteine Metabolism, Glutamate Metabolism, Glycine and Serine Metabolism, Histidine Metabolism, Methionine Metabolism, Threonine and Lysine Metabolism, Tyrosine, Tryptophan, and Phenylalanine Metabolism, and Valine, Leucine, and Isoleucine Metabolism. The Energy Production and Conversion category comprises Citric Acid Cycle, Glycolysis/Gluconeogenesis, and Oxidative Phosphorylation. The Transport, Outer/Inner Membrane category includes Transport, Inner Membrane; Transport, Outer Membrane; and Transport, Outer Membrane Porin.

**Supplementary Figure 7:** Distributions of temperature and pH for database-derived $k_{cat}$ values in the *Escherichia coli* model. Histograms showing the frequency of $k_{cat}$ values retrieved by the WILDkCAT pipeline according to their associated experimental temperatures (left) and pH values (right).

**Supplementary Table 1:** Model annotation requirements for WILDkCAT. COBRApy model fields are classified as mandatory (required for the step to execute), recommended (improves coverage but not strictly required), or not used. For $k_{cat}$ retrieval, at least one of EC code or enzyme UniProt ID must be available (*). For $k_{cat}$ prediction, both enzyme UniProt ID and metabolite KEGG ID are mandatory.

**References:**

1.	Kanehisa, M.; Furumichi, M.; Sato, Y.; Matsuura, Y.; Ishiguro-Watanabe, M. KEGG: Biological Systems Database as a Model of the Real World. *Nucleic Acids Research* **2025**, *53*, D672–D677, doi:10.1093/nar/gkae909.

2.	The UniProt Consortium; Bateman, A.; Martin, M.-J.; Orchard, S.; Magrane, M.; Adesina, A.; Ahmad, S.; Bowler-Barnett, E.H.; Bye-A-Jee, H.; Carpentier, D.; et al. UniProt: The Universal Protein Knowledgebase in 2025. *Nucleic Acids Research* **2025**, *53*, D609–D617, doi:10.1093/nar/gkae1010.

3.	Camborda, S.; Weder, J.-N.; Töpfer, N. CobraMod: A Pathway-Centric Curation Tool for Constraint-Based Metabolic Models. *Bioinformatics* **2022**, *38*, 2654–2656, doi:10.1093/bioinformatics/btac119.

4.	Cock, P.J.A.; Antao, T.; Chang, J.T.; Chapman, B.A.; Cox, C.J.; Dalke, A.; Friedberg, I.; Hamelryck, T.; Kauff, F.; Wilczynski, B.; et al. Biopython: Freely Available Python Tools for Computational Molecular Biology and Bioinformatics. *Bioinformatics* **2009**, *25*, 1422–1423, doi:10.1093/bioinformatics/btp163.

5.	Jensen, F. Activation Energies and the Arrhenius Equation. *Quality & Reliability Eng* **1985**, *1*, 13–17, doi:10.1002/qre.4680010104.

6.	Wang, Z.; Xie, D.; Wu, D.; Luo, X.; Wang, S.; Li, Y.; Yang, Y.; Li, W.; Zheng, L. Robust Enzyme Discovery and Engineering with Deep Learning Using CataPro. *Nat Commun* **2025**, *16*, 2736, doi:10.1038/s41467-025-58038-4.

7.	Kim, S.; Chen, J.; Cheng, T.; Gindulyte, A.; He, J.; He, S.; Li, Q.; Shoemaker, B.A.; Thiessen, P.A.; Yu, B.; et al. PubChem 2025 Update. *Nucleic Acids Research* **2025**, *53*, D1516–D1525, doi:10.1093/nar/gkae1059.

8.	Chang, A.; Jeske, L.; Ulbrich, S.; Hofmann, J.; Koblitz, J.; Schomburg, I.; Neumann-Schaal, M.; Jahn, D.; Schomburg, D. BRENDA, the ELIXIR Core Data Resource in 2021: New Developments and Updates. *Nucleic Acids Research* **2021**, *49*, D498–D508, doi:10.1093/nar/gkaa1025.

9.	Wittig, U.; Rey, M.; Weidemann, A.; Kania, R.; Müller, W. SABIO-RK: An Updated Resource for Manually Curated Biochemical Reaction Kinetics. *Nucleic Acids Research* **2018**, *46*, D656–D660, doi:10.1093/nar/gkx1065.

10.	Chen, Y.; Gustafsson, J.; Tafur Rangel, A.; Anton, M.; Domenzain, I.; Kittikunapong, C.; Li, F.; Yuan, L.; Nielsen, J.; Kerkhoven, E.J. Reconstruction, Simulation and Analysis of Enzyme-Constrained Metabolic Models Using GECKO Toolbox 3.0. *Nat Protoc* **2024**, *19*, 629–667, doi:10.1038/s41596-023-00931-7.

11.	Bekiaris, P.S.; Klamt, S. COBRA-k: A Powerful Framework Bridging Constraint-Based and Kinetic Metabolic Modeling. *Sci. Adv.* **2026**, *12*, eaeb3022, doi:10.1126/sciadv.aeb3022.

12.	Li, F.; Yuan, L.; Lu, H.; Li, G.; Chen, Y.; Engqvist, M.K.M.; Kerkhoven, E.J.; Nielsen, J. Deep Learning-Based Kcat Prediction Enables Improved Enzyme-Constrained Model Reconstruction. *Nat Catal* **2022**, *5*, 662–672, doi:10.1038/s41929-022-00798-z.

13.	Monk, J.M.; Lloyd, C.J.; Brunk, E.; Mih, N.; Sastry, A.; King, Z.; Takeuchi, R.; Nomura, W.; Zhang, Z.; Mori, H.; et al. iML1515, a Knowledgebase That Computes Escherichia Coli Traits. *Nat Biotechnol* **2017**, *35*, 904–908, doi:10.1038/nbt.3956.
